# Rich Beef Tea

**Published:** 1869-12

**Authors:** 


RICH BEEF TEA.—The addition of a small tablespoonful of *cream* to a teacupful of beef tea renders it richer and more nourishing.

